# Betel quid chewing as a risk factor for hepatocellular carcinoma: a case-control study

**DOI:** 10.1054/bjoc.1999.1597

**Published:** 2001-03

**Authors:** J F Tsai, L Y Chuang, J E Jeng, M S Ho, M Y Hsieh, Z Y Lin, L Y Wang

**Affiliations:** Department of Internal Medicine; Biochemistry; Clinical Laboratory, Kaohsiung Medical University College of Medicine; Institute of Biomedical Sciences, Academia Sinica, Taiwan, Republic of China

**Keywords:** betel quid chewing, hepatocellular carcinoma, hepatitis B virus, hepatitis C virus, risk factor

## Abstract

The role of betel quid chewing in the aetiology of hepatocellular carcinoma (HCC) was evaluated in a case–control study including 263 pairs of age- and sex-matched HCC patients and healthy controls. Serum hepatitis B surface antigen (HBsAg), and antibodies to hepatitis C virus (anti-HCV) were determined, and standardized personal interview conducted using a structured questionnaire. Multivariate analysis indicated that betel quid chewing (odds ratio (OR), 3.49; 95% confidence interval (CI), 1.74–6.96), HBsAg (OR, 16.69; 95% CI, 9.92–28.07), anti-HCV (OR, 38.57; 95% CI, 18.15–81.96), and educational duration of less than 10 years (OR, 1.71; 95% CI, 1.05–2.78) are independent risk factors of HCC. In addition, there was an additive interaction between betel quid chewing and chronic infection with either hepatitis B virus (synergy index, 5.37) or hepatitis C virus (synergy index, 1.66). Moreover, risk on HCC increased as duration of betel quid chewing increased, or amount of betel quid consumed (each *P* for trend < 0.0001). © 2001 Cancer Research Campaign http://www.bjcancer.com


*British Journal of Cancer* (2001) **84**(5), 709–713
© 2001 Cancer Research Campaign
doi: 10.1054/ bjoc.2000.1597, available online at http://www.idealibrary.com on IDEAL
http://www.bjcancer.com

### Betel quid chewing as a risk factor for hepatocellular carcinoma: a case–control study

**JF Tsai[1], LY Chuang[2], JE Jeng[3], MS Ho[4], MY Hsieh[1], ZY Lin[1] and LY Wang[1]**

[1]Department of Internal Medicine, [2]Biochemistry, and [3]Clinical Laboratory, Kaohsiung Medical University College of Medicine; [4]Institute of Biomedical Sciences, Academia Sinica, Taiwan, Republic of China

**Summary** The role of betel quid chewing in the aetiology of hepatocellular carcinoma (HCC) was evaluated in a case–control study including 263 pairs of age- and sex-matched HCC patients and healthy controls. Serum hepatitis B surface antigen (HBsAg), and antibodies to hepatitis C virus (anti-HCV) were determined, and standardized personal interview conducted using a structured questionnaire. Multivariate analysis indicated that betel quid chewing (odds ratio (OR), 3.49; 95% confidence interval (CI), 1.74–6.96), HBsAg (OR, 16.69; 95% CI, 9.92–28.07), anti-HCV (OR, 38.57; 95% CI, 18.15–81.96), and educational duration of less than 10 years (OR, 1.71; 95% CI, 1.05–2.78) are independent risk factors of HCC. In addition, there was an additive interaction between betel quid chewing and chronic infection with either hepatitis B virus (synergy index, 5.37) or hepatitis C virus (synergy index, 1.66). Moreover, risk on HCC increased as duration of betel quid chewing increased, or amount of betel quid consumed (each $P$ for trend < 0.0001). © 2001 Cancer Research Campaign http://www.bjcancer.com

**Keywords**: betel quid chewing; hepatocellular carcinoma; hepatitis B virus; hepatitis C virus; risk factor

Hepatocellular carcinoma (HCC) is one of the most common malignant and devastating human tumours in the world (Idilman et al, 1998; DiBisceglie, 1999). Although chronic hepatitis B virus (HBV) and hepatitis C virus (HCV) infection have been implicated as the major risk factors for HCC (Tsai et al, 1996a, 1997a; DiBisceglie, 1999), some HCC occurs in patients without evidence of HBV/HCV infection, suggesting that other environmental or genetic factors, may also be important (Bartsch et al, 1999; DiBisceglie, 1999; Ozturk, 1999). Besides alcohol drinking and cigarette smoking, betel quid chewing is an integral component of the cultural fabric of 10–20% of the human population. It is also a popular habit throughout Taiwan. The cultivation of areca trees and the production of areca nuts increase markedly during the last 3 decades (Ko et al, 1992; Chen and Shaw, 1996). The estimated number of habitual betel quid chewers is around one-tenth of the 20 million inhabitants (Ko et al, 1992). The population of betel quid chewers increased gradually.

The ingredients of betel quid include areca nut (the nut of the *Areca catechu* palm), betel leaf or fruit from *Piper betle*, and red slaked paste. During betel quid chewing, areca nut-derived nitrosamines may methylate and cyanoethylate liver DNA (Prokopczyk et al, 1987), be genotoxic to hepatocytes, and hence produce liver cancer (Bhide et al, 1979; Nishikawa et al, 1992). Areca nut may enhance chemical hepatocarcinogenesis (Bhide et al, 1979; Tanaka et al, 1986). On the other hand, the betel leaf contains high concentrations (15 mg $g^{-1}$ fresh weight) of safrole known to be a rodent hepatocarcinogen (Philips, 1994). Moreover, it has been reported that 37.5% of areca nut samples were infested with aflatoxin B1-producing fungus, *Aspergillus flavus* (Raisuddin and Misra, 1991). Betel quid chewing may therefore have some carcinogenic and tumour-promoting activity in the liver making it pertinent to assess the possible effects of betel quid chewing on the development of HCC, which is one of the most common prevalent cancers in Taiwan (Tsai et al, 1996a, 1997a). The betel quid hypothesis was tested using the data collected in a case-control study of risk factors for HCC

*Received 18 July 2000*
*Revised 27 October 2000*
*Accepted 3 November 2000*

*Correspondence to:* Jung-Fa Tsai, Department of Internal Medicine, Kaohsiung Medical University College of Medicine, 100 Shih-Chuan 1 Rd, Kaohsiung, Taiwan 807, Republic of China

### MATERIALS AND METHODS

### Study population

263 consecutive newly diagnosed patients with HCC, 205 males and 58 females, were recruited as the case group from Department of Internal Medicine, Kaohsiung Medical University Hospital during the period from August 1996 to December 1997. HCC was diagnosed by aspiration cytology or biopsy. During the same study period, 263 healthy community residents, who entered the hospital for physical check-up, matched on age (±3 years) and sex, were enrolled as the control group. All controls had normal serum aminotransferase levels and with no space-occupying lesion in the liver, as evidenced by normal abdominal sonography. There was no difference in median age between cases (59 years; range: 29–83 years) and controls (59 years; range: 29–82 years).

### Structured questionnaire and standardized interview

We designed a structured questionnaire to obtain information on age, sex, educational level, habits of smoking (cigarettes per day and duration of smoking), alcohol drinking (the quantity and duration of drinking, types of alcoholic beverage), betel quid chewing practice (duration of habit, daily amount consumed, type of betel quid ingredients consumed). A habitual betel quid chewer was defined as chewing one quid or more daily for at least one year. A habitual cigarette smoker was defined as smoking one cigarette or more per day for at least one year. A habitual alcohol drinker was defined as drinking alcohol for more than four days a week for at least one year. All HCC cases and matched controls were interviewed by interviewers trained in study details and questionnaire contents.

### Serological examination

An aliquot of 7 ml blood was collected by vacuum syringe with disposable needle from each study subject. Serum samples separated were aliquoted and kept at minus 70˚C until tested. Hepatitis B surface antigen (HBsAg) was tested by radioimmunoassay using commercial kits (Abbott Laboratories, North Chicago, IL). Antibodies to hepatitis C virus (anti-HCV) were detected by second generation ABBOTT HCV EIA (Abbott Laboratories, North Chicago, IL). For anti-HCV, reactive specimens were retested. Only repeatedly reactive specimens were interpreted as anti-HCV positive. Conventional liver function tests were tested by an autoanalyser.

### Statistical analysis

The Mann-Whitney U test was used to compare the difference between medians of continuous variables. The $\chi^2$ test with Yates' correction was used to compare differences between proportions. Odds ratio (OR) with 95% confidence interval (95% CI) was used to estimate causal relations between risk factors and exposure. Mantel extension test for trend was used to estimate the dose–response relationship among risk factors. A conditional logistic regression analysis was used for multivariate analysis. Adjusted odds ratios and 95% CI were derived from logistic regression coefficients to provide an estimate of the statistical association between a given variable and the disease (HCC) with the other variables held constant. Synergy index was used to estimate the interactive effect between risk factors (Rothman, 1986). To calculate the population-attributable risk for factors significantly associated with HCC development in multivariate analysis, the frequency distribution of these risk factors in the control group was used to represent the proportion of persons exposed to the factor in the general population. If there is additive interaction among these risk factors, the sum of each attributable-risk may exceed 100%. An alpha of 0.05 was used as the indicator of significance.

### RESULTS

### Independent risk factors for HCC

Univariate analysis indicated that betel quid chewing (OR = 4.05, 95% CI, 2.35–7.00), HBsAg-positivity (OR = 6.57, 95% CI, 4.38–9.85), anti-HCV-positivity (OR = 9.98, 95% CI, 5.12–19.88), educational level less than 10 years (OR = 1.63, 95% CI, 1.14–2.34), alcohol drinking (OR = 2.41, 95% CI, 1.48–3.94), and smoking (OR = 1.58, 95% CI, 1.09–2.28) were significant risk factors of HCC. The adjusted ORs for factors such as betel quid chewing, HBsAg-positivity, anti-HCV-positivity, and educational level less than 10 years remained significantly elevated even after multivariate analysis (Table 1). The estimated population-attributable risk was 27.63% (95% CI, 13.45–29.84) for subjects with anti-HCV alone, 46.86% (95% CI, 23.41–40.37) for subjects with HBsAg alone, 5.78% (95% CI, 1.08–9.71) for subjects positive for anti-HCV and HBsAg, 20.19% (95% CI, 9.81–23.78) for all betel quid chewers; and 23.22% (95% CI, 9.28–28.41) for those had educational duration of less than 10 years.

### Interactive effect between betel quid chewing and chronic HBV/HCV infection

As shown in Table 2, using subjects without betel quid chewing and negative for both anti-HCV and HBsAg as a referent group, the risk for HCC increased significantly in subjects with HBV and/or HCV infection. The estimated ORs were found to be higher in betel quid chewers infected with HBV or HCV infection (Table 2).

Table 3 displays the interactive effect between betel quid chewing and HCV infection. By using anti-HCV-negative subjects without chewing betel quid as a referent group, either betel quid chewing or presence of anti-HCV were independent risk factors for HCC. The highest ORs were found in anti-HCV-positive betel quid chewers (Table 3). Calculation of synergy index indicated that there was an additive interaction between betel quid chewing and HCV infection. Similarly, the risk for developing HCC was strongly associated with the presence of HBsAg and chewing betel quid (Table 4). Moreover, HBsAg-positive betel quid chewers had the highest OR, and a synergy index of 5.37. This result indicated an additive interaction between betel quid chewing and HBV infection.

### Characteristics of betel quid chewing in HCC patients and controls

All betel quid chewers chewed areca nut. Chewing with betel leaf or with unripe betel fruit was strongly associated with the risk of HCC (Table 5). The duration of chewing betel quid for more than 20 years is an independent risk factor of HCC development (OR = 13.78, 95% CI, 3.88–51.43). Moreover, the longer the duration of betel quid chewing, the higher the risk of developing HCC ($P_{\text{for trend}} < 0.0001$; Table 5).

**Table 1** Univariate and multivariate analyses of risk factors for HCC

| Parameters | Cases (*n* = 263) | Controls (*n* = 263) | OR (95% CI) | Adjusted OR[a] (95% CI) |
|---|---|---|---|---|
| Betel quid chewing | 71 | 22 | 4.05 (2.35–7.00) | 3.49 (1.74–6.96) |
| HBsAg-positive | 171 | 58 | 6.57 (4.38–9.85) | 16.69 (9.92–28.07) |
| Anti-HCV-positive | 85 | 12 | 9.98 (5.12–19.88) | 38.57 (18.15–81.96) |
| Education <10 years | 158 | 126 | 1.63 (1.14–2.34) | 1.71 (1.05–2.78) |

HCC, hepatocellular carcinoma; OR, odds ratio; CI, confidence interval; HBsAg, hepatitis B surface antigen; anti-HCV, antibodies to hepatitis C virus. [a]Derived from conditional logistic regression analysis after adjusting sex, age, habits of alcohol drinking and smoking, and covariates in the table. Only covariates with significant adjusted odds ratio are shown.

*© 2001 Cancer Research Campaign*

**Table 2** Risk of HCC modified by betel quid chewing, status of anti-HCV and HBsAg in HCC patients compared with matched controls

| Betel quid | Status anti-HCV | Status HBsAg | Cases (*n* = 263) | Control (*n* = 263) | Odds ratio (95% CI) |
|---|---|---|---|---|---|
| Nonuser | Negative | Negative | 19 | 180 | 1.0 |
| Nonuser | Negative | Positive | 102 | 50 | 19.32 (10.42–36.17) |
| Nonuser | Positive | Negative | 55 | 7 | 74.43 (27.65–209.70) |
| Nonuser | Positive | Positive | 16 | 4 | 37.89 (10.38–151.37) |
| User | Negative | Negative | 7 | 17 | 3.90 (1.27–11.67) |
| User | Negative | Positive | 50 | 4 | 118.42(35.59–436.95) |
| User | Positive | Negative | 11 | 1 | 104.21 (12.61–351.82) |
| User | Positive | Positive | 3 | 0 | –[a] |

HCC, hepatocellular carcinoma; OR, odds ratio; CI, confidence interval; HBsAg, hepatitis B surface antigen; anti-HCV, antibodies to hepatitis C virus. [a] uncalculable.

**Table 3** Interactions between betel quid chewing and anti-HCV on risk of HCC

| Betel quid chewer | anti-HCV | Cases (*n* = 263) | Controls (*n* = 263) | OR[a] (95% CI) |
|---|---|---|---|---|
| No | Negative | 121 | 230 | 1 |
| No | Positive | 71 | 11 | 12.26 (6.03–25.51) |
| Yes | Negative | 57 | 21 | 5.15 (2.89–9.25) |
| Yes | Positive | 14 | 1 | 26.61 (3.60–116.58) |

HCC, hepatocellular carcinoma; anti-HCV, antibodies to hepatitis C virus; OR, odds ratio; CI, confidence interval. [a]Synergy index = 1.66

**Table 4** Interactions between betel quid chewing and HBsAg on risk of HCC

| Betel quid chewer | HBsAg | Cases (*n* = 263) | Controls (*n* = 263) | OR[a] (95% CI) |
|---|---|---|---|---|
| No | Negative | 74 | 187 | 1 |
| No | Positive | 118 | 54 | 5.52 (3.55–8.59) |
| Yes | Negative | 18 | 18 | 2.52 (1.17–5.42) |
| Yes | Positive | 53 | 4 | 33.48 (11.10–72.69) |

HCC, hepatocellular carcinoma; HBsAg, hepatitis B surface antigen; OR, odds ratio; CI, confidence interval. [a]Synergy index = 5.37

The median value of total amount of betel quid consumed in HCC patients (182 500 quids; range: 73 000–730 000 quids) was higher than that (109 500 quids, range: 10 290–547 500 quids) in controls ($P = 0.0001$). There was an increased risk for developing HCC in subjects consumed more than 100 000 quids (OR = 4.54, 95% CI, 1.40–14.99). There is a positive linear trend between betel quids consumed and the risk for HCC ($P_{\text{for trend}} < 0.0001$; Table 5)

### Clinical characteristics in HCC patients according to betel quid chewing

As shown in Table 6, there was marginal significance in the median age between patients with betel quid chewing and those without ($P = 0.058$). HCC patients with betel quid chewing were predominantly male ($P = 0.0001$), and tended to be anti-HCV-negative ($P = 0.008$). All 71 habitual betel quid chewers were cirrhotics ($P = 0.0001$). Betel quid chewers were frequently habitual smokers ($P = 0.0001$) and alcohol drinkers ($P = 0.0001$).

**Table 5** Risk of hepatocellular carcinoma based on type of betel quid ingredients, duration and total amount of betel quid consumed

| Parameter | Cases | Controls | OR (95% CI) |
|---|---|---|---|
| Type of material in quids | | | |
| Non-user | 192 | 241 | 1.0 |
| Areca-nut with betel leaf | 17 | 6 | 3.55 (1.28–10.30) |
| Areca-nut with betel fruit | 36 | 9 | 5.02 (2.25–11.50) |
| Mixed | 18 | 7 | 3.22 (1.24–8.70) |
| Duration of chewing (years)[a] | | | |
| Non-user | 192 | 241 | 1.0 |
| <20 | 8 | 14 | 0.71 (0.26–1.86) |
| 20–30 | 27 | 5 | 6.77 (2.42–20.46) |
| >30 | 36 | 3 | 15.06 (4.36–39.09) |
| Total amounts consumed (quids × 1000)[b] | | | |
| Non-user | 192 | 241 | 1.0 |
| <100 | 11 | 10 | 1.38 (0.53–3.59) |
| 100–199 | 31 | 7 | 5.55 (2.27–14.17) |
| 200–299 | 15 | 3 | 6.27 (1.67–20.74) |
| >299 | 14 | 2 | 8.78 (1.87–34.01) |

[a,b] $P_{\text{for trend}} < 0.0001$ (Mantel extension test for trend).

**Table 6** Clinical characteristics in patients with hepatocellular carcinoma with regard to betel quid chewing

| | Habitual betel quid chewing Yes (*n* = 71) | No (*n* = 192) | *P* value[a] |
|---|---|---|---|
| Age (years) | 56 (29–81)[b] | 61 (32–83) | 0.05 |
| Educational level | 6 (1–16) | 9 (1–16) | NS |
| Male gender | 67 (94.36)[c] | 138 (71.87) | 0.0001 |
| Cirrhosis | 71 (100) | 142 (73.95) | 0.0001 |
| Smoking | 51 (71.83) | 69 (35.93) | 0.0001 |
| Alcohol drinking | 32 (45.07) | 34 (17.70) | 0.0001 |
| HBsAg-positive | 53 (74.64) | 118 (61.45) | NS |
| Anti-HCV-positive | 14 (19.71) | 71 (36.97) | 0.008 |

NS, nonsignificant. [a]Mann-Whitney U test was used for comparison of continuous data, whereas $\chi^2$ test was used for comparison of proportions. [b]Data were expressed as median (ranges). [c]Data were expressed as number (percentage).

### DISCUSSION

By using a formal epidemiological approach, this study provides evidence that habitual betel quid chewing is an independent risk factors for HCC. However, the estimated population-attributable risks indicate that chronic HBV/HCV infections are the most important risk factors of HCC in Taiwan (Table 1). Since betel quid chewing has not been shown to be a risk factors of HCC before, it is important to validate that our finding is not due to confounding bias. The bias may result from the control selection, information bias, or by un-controlling confounding factor. According to medical records, our healthy controls were healthy subjects who entered the hospital voluntarily for physical check-up. The prevalence of HBsAg (22.1%) and anti-HCV (4.6%) in our healthy controls was similar to those in volunteer blood donors (Tsai et al, 1997b) or community controls in the same area (Tsai et al, 1993, 1996a, 1996b). The estimated prevalence of current betel

© 2001 Cancer Research Campaign 

quid chewers in the same community inhabitants was around 6.5% (Ko et al, 1992; Chen and Shaw, 1996). Moreover, the higher the educational level achieved, the lower the likelihood of being a betel quid chewer (Ko et al, 1992). As the population of betel quid chewing in Taiwan has recently increased year by year, the prevalence of habitual betel quid chewing in our controls (8.36%) seemed reasonable. Among our controls, the frequency of betel quid chewing in subjects with educational level less than 10 years was significantly higher than that in those with more educational level (12.69% vs. 4.37%, $P = 0.024$). Moreover, there was no significant difference in the prevalence rates of habitual alcohol drinking and smoking between our controls and those (11% for alcohol and 65.5% for smoking, respectively) in another case-control study (Chen et al, 1991). Based on the information mentioned above, our controls seem be representative for general population of Taiwan, and make bias unlikely from control selection or under-reporting of life-style habits.

As shown in Tables 3 and 4, although the number of betel quid chewers with either HBV or HCV infection among the controls is small, the OR for HBV- or HCV-infected betel quid chewers seems to be greater than the sum, but lower than the product of the OR for either betel quid chewers alone or subjects with either viral infection alone. Based on a calculation of synergy index, an additive interaction between betel quid chewing and either HBV or HCV infection was deduced (Rothman, 1986). However, there was no multiplicative interaction between betel quid chewing and either HBV or HCV infection on multivariate analysis (data not shown). Taken together, these observations suggest an independent effect and an additive interaction between betel quid chewing and either HBV or HCV infection on the development of HCC.

Both genetic and environmental factors determine individual susceptibility to cancer. Carcinogens derived from betel quid chewing may induce p53 mutation (Wong et al, 1998; Chiang et al, 1999) and over-expression of c-myc protein (Baral et al, 1998) with activated ras oncogene and subsequent over-expression of cell cycle regulatory protein, cyclin D1 (Kuo et al, 1995, 1999). These genetic alterations may have occurred in the process of hepatocarcinogenesis (Idilman et al, 1998; Ozturk, 1999).

Animals with chronic betel quid feeding developed chronic hepatocyte necroinflammation (Sarma et al, 1992) and liver cancer (Bhide et al, 1979; Nishikawa et al, 1992). Although a causal relationship has not been conclusively established, chronic inflammation of the liver appears to be a risk factor for HCC regardless of the underlying aetiology (Tsai et al, 1996a, 1997a; Idilman et al, 1998). Though the mechanism is unknown, episodic necroinflammation has been considered important not only in inducing cirrhosis, but also in promoting transformation and progression to HCC (Idilman et al, 1998). Recent necroinflammation may be a promoting factor that serves as an endogeneous cocarcinogen. Inflammatory byproducts, including oxygen-derived free radicals and other reactive oxygen species, may cause cellular or DNA damage that could be involved in hepatocarcinogenesis (Hagen et al, 1994; Shimoda et al, 1994; Farinati et al, 1999). In this study, all HCC patients with habitual betel quid chewing also had cirrhosis (Table 6). Although cirrhosis is a late sequela of chronic HBV/HCV infection, declining liver function and reactive oxygen species induced during chronic betel quid chewing (Liu et al, 1996) may contribute, at least in part, to an additive interaction between betel quid chewing and chronic HBV/HCV infection.

Little is known about the role of the betel leaf in the betel quid carcinogenesis. The saliva of a betel quid chewer contains on average 420 μmol $l^{-1}$ of safrole (Hwang et al, 1993). Safrole has been classified by the International Agency for Research on Cancer as a group 2B carcinogen (Vainio and Wilbourn, 1992). Experimental study has shown that safrole-induced liver carcinogenesis correlated with the formation of safrole-DNA adducts (Philips, 1994). Recently, safrole-DNA adducts were found in HCC tissue from a heavy betel quid chewer (Liu et al, 2000). The distribution of these adducts was similar to that found in safrole-treated mice: highest in the liver and lower in other tissues. Furthermore, safrole-DNA adduct could not be found in HCC tissue from patients who did not chew betel quid. This information indirectly supports our finding that betel quid chewing is an independent risk factor of HCC. In conclusion, habitual betel quid chewing appears to be an independent risk factor of HCC and an additive interaction between betel quid chewing and chronic HBV/HCV infection.

### ACKNOWLEDGEMENTS

This study was supported in part by the National Science Council of the Republic of China (NSC 85-2331-B-037-084 M14).

### REFERENCES

Bartsch H, Rojas M, Nair U, Nair J and Alexandrov K (1999) Genetic cancer susceptibility and DNA adducts: studies in smokers, tobacco chewers, and coke oven workers. *Cancer Detect Prev* **23**: 445–453

Baral RN, Patnaik S and Das BR (1998) Co-expression of p53 and c-myc proteins linked with advanced stages of betel- and tobacco-related oral squamous cell carcinomas from eastern India. *Eur J Oral Sci* **106**: 907–913

Bhide SV, Shivapurkar NM, Gothoskar SV and Ranadive KJ (1979) Carcinogenicity of betel nut ingredients: feeding mice with aqueous extract and the polyphenol fraction of betel nut. *Br J Cancer* **40**: 922–926

Chen CJ, Liang KY, Chang AS, Chang YC, Lu SN, Liaw YF, Chang WY, Sheen MC and Lin TM (1991). Effects of hepatitis B virus, alcohol drinking, cigarette smoking and familial tendency on hepatocellular carcinoma. *Hepatology* **13**: 398–406.

Chen JW and Shaw JH (1996) A study on betel quid chewing behavior among Kaohsiung residents aged 15 years and above. *J Oral Pathol Med* **25**: 140–143

Chiang CP, Huang JS, Wang JT, Liu BY, Kuo YS, Jahn LJ and Kuo YP (1999) Expression of p53 protein correlates with decreased survival in patients with areca quid chewing and smoking-associated oral squamous cell carcinoma in Taiwan. *J Oral Pathol Med* **28**: 72–76

DiBisceglie AM (1999). Malignant neoplasms of the liver. In: *Schiff's disease of the liver*. 8th edn. Schiff ER, Sorrell MF, Maddrey WC, (eds) pp. 1281–1304. Lippincott-Raven Publishers: Philadelphia

Farinati F, Cardin R, Degan P, De Maria N, Floyd RA, Van Thiel DH and Naccarato R (1999) Oxidative DNA damage in circulating leukocytes occurs as an early event in chronic HCV infection. *Free Radic Biol Med* **27**: 1284–1291

Hagen TM, Huang S, Curnutte J, Fowler P, Martinez V, Wehr CM, Ames BN and Chisari FV (1994) Extensive oxidative DNA damage in hepatocytes of transgenic mice with chronic active hepatitis destined to develop hepatocellular carcinoma. *Proc Natl Acd Sci USA*. **911**: 12808–12828

Hwang LS, Wang CK, Sheu MJ and Kao LS (1993) Phenolic compounds of piper betle flower as flavoring and neuronal activity modulating agents. In *Food phytochemicals for cancer prevention*. Ho CT, Osawa T, Huang MT, Rossen RT (eds) pp. 186–191. American Chemical Society: Washington, DC

Idilman R, De Maria N, Colantoni A and Van Thiel DH (1998) Pathogenesis of hepatitis B and C-induced hepatocellular carcinoma. *J Viral Hepatitis* **5**: 285–299

Ko YC, Chiang TA, Chang SJ and Hsieh SF (1992) Prevalence of betel quid chewing habit in Taiwan and related sociodemographic factors. *J Oral Pathol Med* **21**: 261–264

Kuo MYP, Chang HH, Hahn LJ, Wang JT and Chiang CP (1995) Elevated ras p21 expression in oral premalignant lesions and squamous cell carcinoma in Taiwan. *J Oral Pathol Med* **24**: 255–260

Kuo MYP, Lin CY, Hahn LJ, Cheng SJ and Chiang CP (1999) Expression of cyclin D1 is correlated with poor prognosis in patients with areca quid

 © 2001 Cancer Research Campaign chewing-related oral squamous cell carcinomas in Taiwan. *J Oral Pathol Med* **28**: 165–169
Liu TY, Chen CL and Chi CW (1996). Oxidative damage to DNA induced by areca nut extract. *Mutation Res* **367**: 25–31
Liu CJ, Chen CL, Chang KW, Chu CH and Liu TY (2000) Safrole in betel quid may be a risk factor for hepatocellular carcinoma: case-report. *CMAJ* **162**: 359–360.
Nishikawa A, Prokopczyk B, Rivenson A, Zang E and Hoffmann D (1992). A study of betel quid carcinogenesis – VIII. Carcinogenicity of 3 (methylnitrosamino) propionaldehyde in F344 rats. *Carcinogenesis* **13**: 369–372
Ozturk M (1999). Genetic aspects of hepatocellular carcinogenesis. *Semin Liver Dis* **19**: 235–242
Philips DH (1994) DNA adducts derived from safrole, estragole and related compounds, and from benzene and its metabolites. In: *DNA adducts: identification and biological significance*. Hemminki K, Dipple A, Shuker DEG, Kadlubar FF, Segerback D, Bartsch H, (eds) pp. 131–140. International Agency for Research on Cancer Research: Lyon, France
Prokopczyk B, Rivenson A, Bettinato P, Bertinato P, Brunnemann D and Hoffmann D (1987) 3-(Methylnitorsamino)propionitrile: Occurrence in saliva of betel quid chewers, carcinogenicity, and DNA methylation in F344 rats. *Cancer Res* **47**: 467–471
Raisuddin S and Misra JK (1991) Aflatoxin in betel nut and its control by use of food preservatives. *Food Add Contam* **8**: 707–712
Ramchandani AG, D'Souza AV, Borges AM and Bhisey RA (1998) Evaluation of carcinogenic/co-carcinogenic activity of a common chewing product, pan masala, in mouse skin, stomach and esophagus. *Int J Cancer* **75**: 225–232.
Rothman KJ (1986) *Modern epidemiology*, pp. 311–326. Little, Brown & Company: Boston
Sarma AB, Chakrabarti J, Chakrabarti A, Banerjee TS, Roy D, Mukherjee D and Mukherjee A (1992) Evaluation of pan masala for toxic effects on liver and other organs. *Food Chem Toxicol* **30**: 161–163
Shimoda R, Nagashima M, Sakamoto M, Yamaguchi N, Hirohashi S, Yokota J and Kasai H (1994) Increased formation of oxidative DNA damage, 8-hydroxydeoxyguanosine, in human livers with chronic hepatitis. *Cancer Res* **543**: 3171–3172
Tanaka T, Kuniyasu T, Shima H, Sugie S, Mori H, Takahashi M, and Hirono I (1986) Carcinogenicity of betel quid. III. Enhancement of 4-nitroquinoline-1-oxide- and N-2-fluorenylacetamide-induced carcinogenesis in rats by subsequent administration of betel nut. *J Natl Cancer Inst* **77**: 777–781
Tsai JF, Chang WY, Jeng JE, Ho MS, Wang LY, Hsieh MY, Chen SC, Chuang WL, Lin ZY and Tsai JH (1993) Hepatitis C virus infection as a risk factor for nonalcoholic cirrhosis in Taiwan. *J Med Virol* **41**: 296–300
Tsai JF, Jeng JE, Ho MS, Chang WY, Hsieh MY, Lin ZY and Tsai JH (1996a) Additive effect modification of hepatitis B surface antigen and e antigen on the development of hepatocellular carcinoma. *Br J Cancer* **73**: 1498–1502
Tsai JF, Jeng JE, Ho MS, Chang WY, Lin ZY and Tsai JH (1996b). Independent and additive effect modification of hepatitis C and B viruses infection on the development of chronic hepatitis. *J Hepatol* **24**: 271–276.
Tsai JF, Jeng JE, Ho MS, Chang WY, Hsieh MY, Lin ZY and Tsai JH (1997a) Effect of hepatitis C and B virus infection on risk of hepatocellular carcinoma: a prospective study. *Br J Cancer* **76**: 968–974
Tsai JF, Jeng JE, Ho MS, Wang CS, Chang WY, Hsieh MY, Lin Z and Tsai JH (1997b) Serum alanine aminotransferase level in relation to hepatitis B and C virus infections among blood donors. Liver **17**: 24–29.
Vainio H and Wilbourn J (1992) Identification of carcinogenesis within the IARC monograph program. *Scand J Work Environ Health* **18** (suppl 1): 64–73
Wong YK, Liu TY, Chang KW, Lin SC, Chao TW, Li PL and Chang CS (1998) p53 alterations in betel quid- and tobacco-associated oral squamous cell carcinomas from Taiwan. *J Oral Pathol Med* **27**: 243–248

*© 2001 Cancer Research Campaign*
